# Indicators of maladaptive emotions in patients with cancer as assessed by oncologists and nurses

**DOI:** 10.1007/s00520-025-09917-5

**Published:** 2025-10-11

**Authors:** G. Elise Doppenberg-Smit, Myra E. van Linde, Femke Lamers, Adriaan W. Hoogendoorn, Annemarie M. J. Braamse, Evelien J. M. Kuip, Mariette Labots, Rune A. W. van de Wetering, Inge M. Werter, Aartjan T. F. Beekman, Henk M. W. Verheul, Joost Dekker

**Affiliations:** 1 Department of Psychiatry, Amsterdam University Medical Center, Vrije Universiteit Amsterdam, Amsterdam, Netherlands; 2 Mental Health Program, Amsterdam Public Health, Amsterdam, the Netherlands; 3 Cancer Treatment and Quality of Life, Cancer Centre Amsterdam, Amsterdam, the Netherlands; 4 Department of Medical Oncology, Amsterdam University Medical Center, Vrije Universiteit Amsterdam, Amsterdam, the Netherlands; 5 Department of Medical Psychology, Amsterdam University Medical Center, University of Amsterdam, Amsterdam, the Netherlands; 6 Department of Medical Oncology, Radboud Medical Center, Nijmegen, the Netherlands; 7 Department of Anesthesiology, Pain and Palliative Care, Radboud Medical Center, Nijmegen, the Netherlands; 8 Department of Medical Oncology, Slingeland Ziekenhuis, Doetinchem, the Netherlands; 9 Department of Medical Oncology, Rijnstate Hospital, Arnhem, the Netherlands; 10 Department of Medical Oncology, Erasmus Medical Center, Rotterdam, the Netherlands

**Keywords:** Cancer, Emotion, Symptom, Mental disorder, Mental health

## Abstract

**Purpose:**

The clinical management of patients with cancer by oncologists and nurses needs to differentiate between maladaptive emotions that do require professional mental health care and adaptive emotions that do not require such care. Previous qualitative research identified six symptoms as potential indicators of maladaptive emotions. These included lingering, increasing or extreme emotions, emotions that interfere with daily life or with treatment, and unexplained somatic symptoms. The present study tested the validity of these symptoms as indicators of maladaptive emotions in patients with cancer.

**Methods:**

Patients with a solid malignancy were assessed 3 to 7 months after the start of chemo- or immunotherapy. Patients were categorized as experiencing either adaptive or maladaptive emotions, using two reference standards: (a) a psychiatric diagnostic assessment or (b) patient’s subjective need for professional mental health care. Oncologists and nurses assessed the presence of the six symptoms, using a checklist. A total count was made of the number of symptoms that were rated as present.

**Results:**

As hypothesized, the total symptom count was statistically significantly greater in patients with maladaptive emotions than in patients with adaptive emotions. This was consistently observed for the ratings by oncologists as well as nurses, and for both reference standards.

**Conclusion:**

The current study confirmed six symptoms assessed by oncologists and nurses as valid indicators of maladaptive emotions in patients with cancer. We recommend assessing these symptoms in the broader context of managing emotional well-being of patients with cancer.

**Supplementary Information:**

The online version contains supplementary material available at 10.1007/s00520-025-09917-5.

## Introduction

Oncology care is multidisciplinary and multifaceted in nature, addressing many aspects of patients’ health [1–3]. Medical oncologists and nurses play a key role in the multidisciplinary team. In addition to providing medical-somatic care, they promote patients’ well-being [1–5], addressing distress in the psychological (i.e. cognitive, behavioural, emotional), social, spiritual and physical domains [6]. In the emotional domain, this includes (i) providing emotional support and (ii) identifying patients in need of professional mental health care and providing a referral [7, 8].

A number of studies suggested that oncologists and nurses are not sufficiently sensitive to the emotional needs of their patients. These studies reported a low sensitivity for identifying emotional symptoms, leaving a considerable number of patients with unidentified emotional needs [9–11]. However, these studies did not differentiate between adaptive and maladaptive emotions. Emotions are a natural and potentially adaptive response to a life-threatening disease and a burdensome treatment [7, 12]. Emotions alert, motivate and prepare patients to deal with these events [13]. For example, fear causes cognitive, behavioural and physiological changes that help a person face a threatening event [14]. Such emotions are *adaptive*: they help patients to cope with the threats and challenges posed by cancer. Emotions can also be *maladaptive*, in the sense that they are disproportionally severe or persistent and that they interfere with functioning [7]. For example, a major depressive disorder may keep a patient from attending treatment. Maladaptive emotions interfere with patients’ attempts to cope with cancer. Thus, a distinction can be made between adaptive and maladaptive emotions in patients with cancer [12].

We have argued that adaptive and maladaptive emotions are associated with a differential need for care [7, 15]. Presumably, patients with adaptive emotions prefer to deal with emotions on their own or with support from relatives, friends, peers and caregivers (i.e. doctors and nurses), but outside professional mental health care. There is considerable evidence that patients prefer to deal with their cancer-related emotions on their own [16–21]. For example, Clover et al. [17] reported that most patients with significant distress declined help, with the most common reason being ‘I prefer to manage myself’. Patients welcome emotional support from relatives, friends, peers and primary caregivers (i.e. doctors and nurses), but frequently do not want a referral to professional mental health care (provided by a psychotherapist, psychologist or psychiatrist). We suppose that it is patients with adaptive emotions who take this attitude [7]. In contrast, in case of maladaptive emotions professional mental health care may be indicated: these patients are unable to resolve emotional disorders on their own, whereas professional care can help [7]. Indeed, patients with more severe distress have been found to prefer professional care [17, 20, 21]. Of course, the link between adaptive/maladaptive emotions and the need for professional mental health care is not absolute: patients with adaptive emotions may also have a need to talk to a mental health professional. Still, the studies cited above support the premise that adaptive and maladaptive emotions are associated with a differential need for care.

In a study on the clinical assessment of emotions by oncologists and nurses, we used the distinction between adaptive and maladaptive emotions and the associated differential need for professional mental health care [22]. Oncologists and nurses were found to be sensitive to emotional disorders in their patients, defined as maladaptive emotions that did require professional mental health care. The sensitivity to maladaptive emotions requiring professional mental health care was found to be 0.85 (95% CI: 0.64–0.95) [22]. Subsequently, we examined how oncologists and nurses identify patients with maladaptive emotions requiring professional mental health care, in a qualitative study [23]. They reported using a strategy that allowed patients time to adjust to their cancer diagnosis, while monitoring patients’ psychological well-being, especially when a patient exhibited specific risk factors. Perceived risk and protective factors for emotional problems included personal, social and disease- and treatment-related factors (e.g. a history of emotional problems, little social support or extreme side effects). Oncologists and nurses considered referral for professional mental health care when they noticed specific indicators of emotional disorders. These presumed indicators of maladaptive emotions and the resulting need for professional mental health care included the following symptoms: lingering or increasing emotions, a disproportionate intensity of emotions, emotions with a negative impact on a patient’s daily life or treatment, and unexplained somatic symptoms that were potentially triggered by emotional problems [23].

The present study tested the validity of these symptoms assessed by oncologists and nurses as indicators of maladaptive emotions in patients with cancer. We hypothesized that oncologists and nurses were more likely to rate these symptoms as present in patients with maladaptive emotions compared to patients with adaptive emotions.

## Methods

### Design

This was a cross-sectional, observational study that was part of a parent study on adaptive and maladaptive emotions in patients with cancer [24]. Patients with a solid malignancy, either non-metastatic disease or metastatic disease, were assessed 3 to 7 months after the start of chemo- or immunotherapy. Patients were categorized as experiencing either adaptive or maladaptive emotions using one of two reference standards: (a) a psychiatric diagnostic assessment or (b) a patient’s subjective need for professional mental health care. Oncologists and nurses assessed the presence of six symptoms hypothesized to be an indicator of maladaptive emotions. The study analyzed whether oncologists and nurses were more likely to rate these symptoms as present in patients with maladaptive emotions compared to patients with adaptive emotions.

### Patients, oncologists and nurses

Patients were recruited in two academic and four non-academic hospitals in the Netherlands. Inclusion criteria were diagnosis of a solid malignancy cancer, non-metastatic disease or metastatic disease; between 3 and 7 months after start of treatment with (neo)adjuvant or first-line chemotherapy or with (neo)adjuvant or first-line immunotherapy; life expectancy of more than 3 months. Exclusion criteria were age < 18; insufficient command of the Dutch language; severe psychopathology, other than a mood or anxiety disorder (e.g. lifetime history of bipolar disorder, schizophrenia, major depressive disorder with psychotic features, schizoaffective disorders); severe cognitive impairments (e.g. dementia); (indication of) brain tumor or –metastases; history of severe drug or alcohol abuse within the past 6 months; not competent in the use of a mobile phone; or no informed consent.

As patients are more likely to experience adaptive than maladaptive emotions, disproportional random sampling was applied to increase the inclusion of patients who experienced maladaptive emotions. After the inclusion of 70 patients with adaptive emotions, eligible patients were first screened with the Extended version of the Kessler-10 questionnaire (EK10) [25, 26] and patient’s subjective need for professional mental health care[22]. Only patients who were considered highly likely to experience maladaptive emotions were invited for further assessments.

Patients’ medical records were reviewed to see which oncologists and nurses were involved in the patient’s treatment. An oncologist and nurse with recent patient contact were asked to rate the six symptoms. The assessment was conducted as soon as possible after they consulted with the patient.

### Measures

#### Symptoms

On a checklist, oncologist and nurses rated whether the patient had shown one or more of six symptoms in the past 3 months to such an extent that professional mental health care was (probably) necessary in their opinion. The symptoms on the checklist are shown in Box 1. Examples of each of the symptoms were provided. The oncologists and nurses could indicate either ‘yes’ or ‘no’, with an explanatory note if needed. In four hospitals, oncologists delegated the role of patients’ primary caretaker to a nurse specialist: in these cases the nurse specialists’ checklist was considered the oncologist’s checklist. A total count was made of the number of symptoms that were rated as present.

#### Maladaptive and adaptive emotions

Two reference standards were used to categorize patients as having either adaptive or maladaptive emotions: (i) a psychiatric diagnostic assessment or (ii) patient’s subjective need for professional mental health care.

*(i) Psychiatric diagnostic assessment.* The Composite International Diagnostic Interview (CIDI) section Anxiety and Depression was used to determine the presence of a major depressive disorder or anxiety disorder in the past month according to DSM-IV criteria [27, 28]. The interview was conducted by a trained researcher (ED) or a trained research assistant. Adjustment disorder was assessed with the self-reported Four Dimensional Symptoms Questionnaire (4DSQ): patients who scored above the validated cut-off of 20 on the section Distress were categorized as having an adjustment disorder [29]. Maladaptive emotions were rated as present if the patient had a positive score for a major depressive disorder, anxiety disorder (panic disorder, agoraphobia without panic, specific phobia, social phobia, generalized anxiety disorder or posttraumatic stress disorder) or an adjustment disorder in the past month. Patients without a current diagnosis on the CIDI and a score below the cut-off on the 4DSQ were categorized as having adaptive emotions.

*(ii) Patient’s subjective need for professional mental health care.* Patients filled out the Problem List where they could indicate to what extent they had experienced 10 emotional problems in the past three months (not at all, somewhat, rather, much, very much) [30]. When one or more emotional problems were rated as at least somewhat present, they indicated whether they had received or wished to receive a referral to a professional for those emotional problems. Patients were categorized as having maladaptive emotions when they received or wished to have such a referral [22, 31]. Other patients were categorized as having adaptive emotions [22, 31].

#### Background characteristics and medical diagnoses

Sociodemographic information (age and gender) was selfreported, as well as professional mental health care in the past year. Medical information regarding diagnosis and disease stage was retrieved from the medical file.

**Figure:**
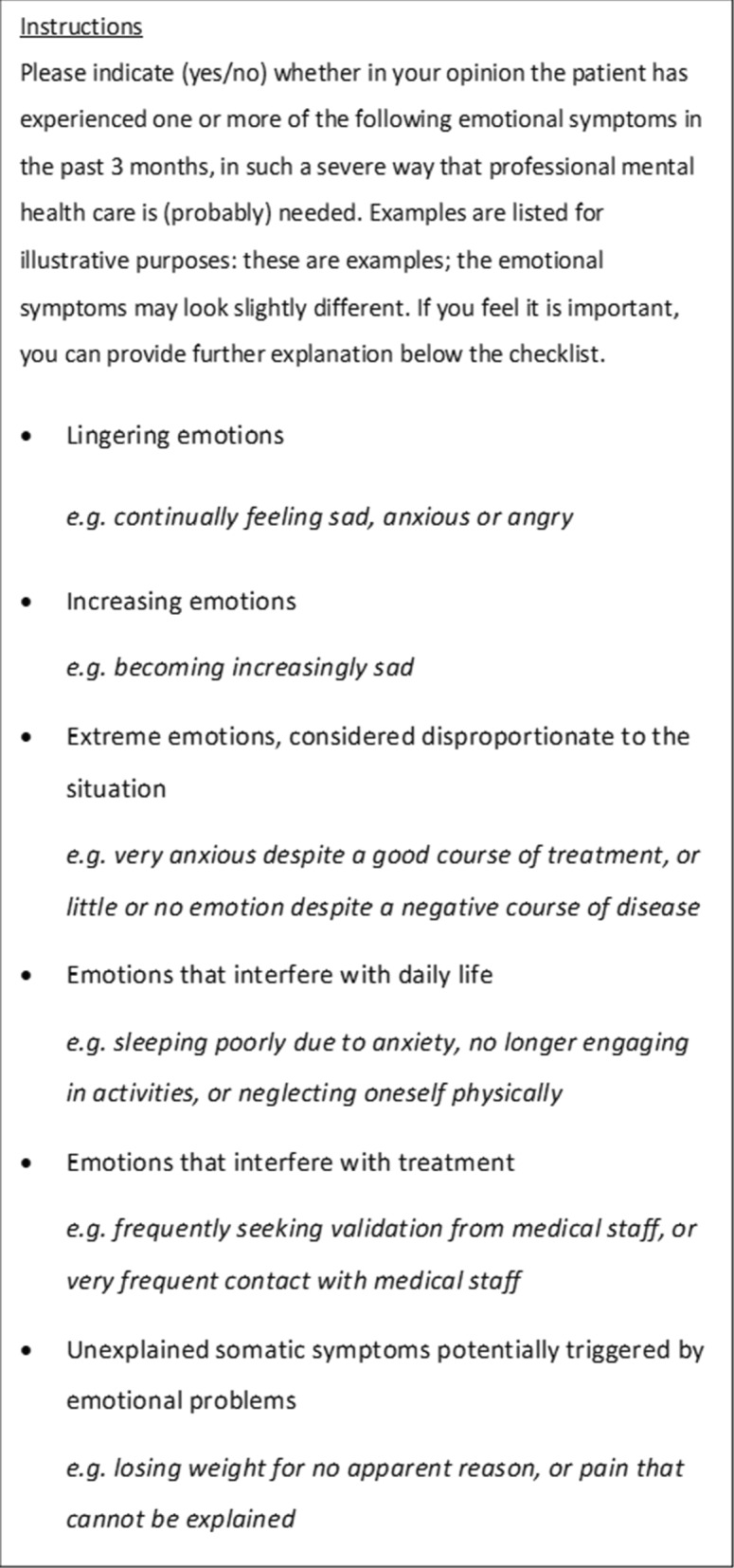
**Box 1** Symptoms presumably indicating maladaptive emotions

### Statistical analyses and power analysis

Descriptive statistics on the presence of symptoms were calculated for the separate symptoms and the total count. To compare the presence of symptoms in the maladaptive versus adaptive group, a Welch Two Sample t-test was conducted on the total count of the six symptoms, for oncologists and nurses separately. These analyses were conducted twice, once using the psychiatric diagnostic assessment as reference standard, and once using patient’s subjective need for help as reference standard. Cohen’s* d* was calculated to estimate the magnitude of the effect. All analyses were conducted in R version 4.2.1.

Originally, the plan was to perform separate analyses in each disease-stage stratum (non-metastasized or metastasized disease). The aim was to recruit 40 patients with adaptive emotions and 40 patients with maladaptive emotions in each stratum. According to the power analysis, with an alpha = 0.05 and power = 0.80, the study would be able to detect a medium to large effect size of d = 0.63. However, because fewer than planned patients with maladaptive emotions were included and because the adaptive and maladaptive groups had a similar distribution of patients with non-metastatic or metastatic disease, as well as a similar mean total symptom count (see Results section), the analyses were performed in the combined group of patients with non-metastasized or metastasized disease.

## Results

### Patients, oncologists and nurses

Of the 134 patients who participated in the parent study, 132 had a checklist on symptoms completed by either their oncologist or nurse and were included in the present study. The mean age was 57.6 years (range 22–86 years) and 58.3% were female (see Table 1). Seventy-five percent of the patients had been diagnosed with gastro-intestinal cancer, breast cancer, or melanoma, and patients with non-metastatic disease comprised 56.1% of the sample. The majority (75.8%) had not received professional mental health care from a psychiatrist, psychologist or social worker in the past year. In these patients, 40 oncologists, 19 nurse specialists, and 78 nurses rated the six symptoms.

**Table 1:** Patient characteristics

	Mean (SD) or *n* (%)
Age	57.6 (12.8)
Gender	
Female	77 (58.3)
Male	55 (41.7)
Diagnosis	
Gastro-intestinal cancer	34 (25.8)
Breast cancer	33 (25.0)
Melanoma	32 (24.2)
Urogenital cancer	12 (9.1)
Gynaecological cancer	8 (6.1)
Sarcoma	7 (5.3)
Head/neck cancer	4 (3.0)
Other	2 (1.5)
Metastatic disease	
Yes	58 (43.9)
No	74 (56.1)
Professional mental health care in past year	
Yes	32 (24.2)
No	100 (75.8)

Total number of patients = 132

### Adaptive or maladaptive emotions

Around one quarter of patients were categorized as having maladaptive emotions and three-quarters as having adaptive emotions. This result was obtained for both reference standards (the psychiatric diagnostic assessment and patient’s subjective need for professional mental health care; see Table 2). There was fair agreement in categorization based on the two reference standards (kappa = 0.30)[32]. The adaptive and maladaptive group did not differ in the male to female ratio (psychiatric diagnostic assessment: *X*^2^(1, *N* = 132) = 0.12*, p* = 0.73; patient’s subjective need for professional mental health care: *X*^2^(1, *N* = 132*)* = 1.52, *p* = 0.22) or in the ratio of patients with non-metastatic or metastatic disease (psychiatric diagnostic assessment: *X*^2^(1, *N* = 132) = 0.03*, p* = 0.86; patient’s subjective need for professional mental health care: *X*^2^(1, *N* = 132) = 0.12, *p* = 0.73).

**Table 2:** Crosstabulation of adaptive and maladaptive emotions according to two reference standards: number of patients (%)

		Patient’s subjective need for professional mental health care		
		Adaptive	Maladaptive	Total
Psychiatric diagnostic assessment	Adaptive	81 (61.4%)	19 (14.4%)	100 (75.8%)
	Maladaptive	16 (12.1%)	16 (12.1%)	32 (24.2%)
	Total	97 (73.5%)	35 (26.5%)	132 (100%)

### Symptoms

Oncologist-completed symptom checklists were available for 129 patients, while nurse-completed checklists were available for 90 patients. The lower completion rate for nurses was mostly due to the lack of recent patient contact. Emotions interfering with daily life, lingering emotions and increasing emotions were mentioned most frequently (see Appendix), while extreme emotions, unexplained somatic symptoms and emotions interfering with treatment were mentioned less frequently. At least one of the six symptoms was rated as present in 28.7% and 25.6% of patients by oncologists and nurses, respectively. The mean total symptom count was 0.53 (*SD* = 0.98) for oncologists and 0.42 (SD 0.92) for nurses (see Table 3 for further details). The total symptom count was similar in patients with non-metastatic or metastatic disease (oncologists: *t*(127) = 0.09, *p* = 0.93; nurses: *t*(88) = 1.02, *p* = 0.31).

**Table 3:** Total count of symptoms rated present by oncologist or nurse in patients with adaptive or maladaptive emotions

		Number of patients	Median	Mean (95% CI)	SD	*t*-test, *p*-value	Effect size
Reference standard: psychiatric diagnostic assessment							
Oncologist	Adaptive emotions	97	0	0.33 (0.18, 0.48)	0.73	*t*(37.22) = 3.31,	*d* = 0.90
	Maladaptive emotions	32	1	1.16 (0.67, 1.64)	1.35	*p* = 0.002	
	Total	129	0	0.53 (0.36, 0.71)	0.98		
Nurse	Adaptive emotions	68	0	0.21 (0.08, 0.34)	0.53	*t*(22.89) = 2.81,	*d* = 1.04
	Maladaptive emotions	22	1	1.09 (0.45, 1.74)	1.44	*p* = 0.01	
	Total	90	0	0.42 (0.23, 0.62)	0.92		
Reference standard: patient’s subjective need for professional mental health care							
Oncologist	Adaptive emotions	94	0	0.39 (0.24, 0.55)	0.77	*t*(42.29) = 2.14,	*d* = 0.54
	Maladaptive emotions	35	0	0.91 (0.45, 1.38)	1.36	*p* = 0.04	
	Total	129	0	0.53 (0.36, 0.71)	0.98		
Nurse	Adaptive emotions	62	0	0.24 (0.08, 0.40)	0.62	*t*(32.60) = 2.24,	*d* = 0.65
	Maladaptive emotions	28	0	0.82 (0.31, 1.33)	1.31	*p* = 0.03	
	Total	90	0	0.42 (0.23, 0.62)	0.92		

(1) Patients were categorized as experiencing either adaptive or maladaptive emotions, using two reference standards: (a) psychiatric diagnostic assessment or (b) patient’s subjective need for professional mental health care. (2) Total count of symptoms had a theoretical range from 0 to 6. (3) Welch’s *t*-test and effect size results concern the comparison of the group of patients with adaptive emotions versus the group of patients with maladaptive emotions

### Symptoms in patients with adaptive or maladaptive emotions

The separate symptoms were more frequently rated as present in patients with maladaptive emotions compared to patients with adaptive emotions (there were only two exceptions among a total of 24 comparisons; see Appendix). This pattern was observed for both oncologists and nurses, and for both the psychiatric diagnostic assessment and patient’s subjective need for professional mental health care as reference standard. Due to the low count of separate symptoms, differences could not be tested statistically. However, the differences on the total symptom count could be tested (see below and Table 3).

### Psychiatric diagnostic assessment as reference standard

#### Oncologists

Regarding the psychiatric diagnostic assessment as the reference standard, oncologists rated patients with maladaptive emotions as having a greater total symptom count than patients with adaptive emotions (*n* = 129; *t*(37.22) = 3.31, *p* < 0.01, *d* = 0.90). This was a large effect.

#### Nurses

A similar pattern was observed for symptoms rated by nurses, who rated patients with maladaptive emotions as having a greater total symptom count than patients with adaptive emotions (*n* = 90; *t*(22.89) = 2.81, *p* = 0.01, *d* = 1.04). This was a large effect.

### Patient’s subjective need for professional mental health care as reference standard

#### Oncologists

With patient’s subjective need for professional mental health care as reference standard, patients with maladaptive emotions had a greater total symptom count rated by oncologists than patients with adaptive emotions (*n* = 129; *t*(42.29) = 2.14, *p* = 0.04, *d* = 0.54). This was a moderate effect.

#### Nurses

Similarly, nurses rated patients with maladaptive emotions as having a greater total symptom count than patients with adaptive emotions (*n* = 90; *t*(32.60) = 2.24, *p* = 0.03, *d* = 0.65). This was a moderate effect.

## Discussion

The present study tested the validity of six symptoms assessed by oncologists and nurses as indicators of maladaptive emotions in patients with cancer. As hypothesized, the total count of symptoms was statistically significantly greater in patients with maladaptive emotions than in patients with adaptive emotions. This outcome was consistently observed for ratings by oncologists as well as nurses, and for the reference standards psychiatric diagnostic assessment as well as patient’s subjective need for professional mental health care. Effect sizes were moderate to large (Cohen’s d ranging from 0.54 to 1.04). Individual symptoms showed largely the same results. These results confirm that the six symptoms assessed by oncologists and nurses are valid indicators of maladaptive emotions in patients with cancer.

Approximately a quarter of patients were categorized as having maladaptive emotions and three-quarters as having adaptive emotions. Concerning the dimension adaptive/maladaptive emotions, the present sample of patients cannot be considered representative due to the oversampling of patients with maladaptive emotions. Nevertheless, the final distribution is consistent with estimates in more representative studies [7, 33]. Similarly, in approximately one quarter of patients at least one of the six symptoms was rated as present, although some individual symptoms were rated less frequently. Emotions interfering with daily life, lingering emotions and increasing emotions were mentioned most frequently.

Conceptually, a psychiatric diagnosis and patient’s subjective need for professional mental health care are two different entities. Empirically, this was confirmed in the present study, with only a fair agreement in categorization based on the two reference standards. Nevertheless, the results with regard to the six symptoms were similar, which can be interpreted as evidence for the robustness and generalizability of these six symptoms as indicators of maladaptive emotions. The validity of these indicators does not appear to depend on one specific reference standard but holds for both reference standards. Our hypothesis was confirmed in the case of both oncologists and nurses. This suggests that both of these professional groups are able to competently assess the six symptoms.

Nevertheless, some methodological considerations need to be addressed. First, patients were assessed 3 to 7 months after the start of chemo- or immunotherapy. We consider this a major strength of the study: we avoided the immediate, potentially less stable emotional responses directly after diagnosis. Clinical experience suggests that after 3 to 7 months the differentiation between patients with adaptive or maladaptive emotions is rather stable. Second, the reference standards (the psychiatric diagnostic assessment and patient’s subjective need for professional mental health care) each encompass many more aspects of mental dysfunction than just the six symptoms considered here. A one-to-one relationship between the reference standards and the six symptoms is therefore not to be expected. Yet even with these non-ideal reference standards, our hypothesis was confirmed. Nevertheless, we recommend to develop better indicators of maladaptive emotions. Third, fewer patients with maladaptive emotions were recruited than originally expected. We planned a longer recruitment period, as maladaptive emotions are less prevalent than adaptive emotions [7]. Nevertheless, we were unable to recruit the planned number of patients with maladaptive emotions. These patients may be less inclined to participate in research or oncologists may be less inclined to invite these patients to participate in research. In order to have a sufficient number of patients with maladaptive emotions, we decided to merge patients with non-metastasized and metastasized disease. This decision is unlikely to have resulted in analysis bias because patients with non-metastasized or metastasized disease showed the same distribution of adaptive or maladaptive emotions, and a similar mean total symptom count. Fourth, the checklist as completed by nurses was missing for almost a third of patients, which was mainly due to a lack of recent patient contact in the outpatient clinic.

The six symptoms considered here were originally identified in a qualitative study [23]. The present quantitative study confirms that these symptoms are indeed indicators of maladaptive emotions in patients with cancer. However, we advise against using the symptoms as stand-alone indicators of maladaptive emotions. Instead, we recommend using these symptoms in the context of a broader strategy for managing emotions in patients with cancer. We have previously recommended ‘emotional support and case finding’ as the preferred approach to the management of emotions in patients with cancer [7]. The two main principles of that approach are: (1) Emotional support: (a) The treating team, consisting of doctors, nurses and allied health staff, is responsive to the emotional needs of patients with cancer and provides emotional support, and (b) the treating team provides information on external sources of emotional support. (2) Case finding: The treating team identifies patients in need of mental health care by means of case finding and provides a referral to mental health care as indicated. For case finding, oncologists and nurses have been found to use the strategy of allowing patients time to adjust while monitoring psychological well-being, especially when specific risk factors are present [23]. Within the context of this strategy, we recommend using the six symptoms discussed here to help identify patients with maladaptive emotions and an accompanying need for professional mental health care. In addition, although their own clinical experience is crucial, oncologists and nurses may need to develop their competencies in this respect. We recommend mental health professionals to provide training and coaching to oncologists and nurses to enhance the required competencies in this regard [34].

## Conclusion

The present study confirmed six symptoms assessed by oncologists and nurses as valid indicators of maladaptive emotions in patients with cancer. We recommend assessing these symptoms in the broader context of managing emotional well-being of patients with cancer.

## Supplementary Information

Below is the link to the electronic supplementary material.

ESM1 (DOCX 21.1 KB)

## Data Availability

Data are available upon reasonable request.

## Declarations

### Study registration

NCT05062447

First posted: 2021-09-30
