# Supplementary material for: Indicators of maladaptive emotions in patients with cancer as assessed by oncologists and nurses
Source: Support Care Cancer. 2025 Oct 11;33(11):930. doi: 10.1007/s00520-025-09917-5 (PMC12513930; doi:10.1007/s00520-025-09917-5)
Supplement: Supplementary file 1 — (DOCX 21.1 KB) [file 520_2025_9917_MOESM1_ESM.docx]

**Appendix A.** Separate symptoms in patients with adaptive or maladaptive emotions, rated by oncologist or nurse

|  | Number of  patients | Lingering emotions  n (%) | Increasing emotions  n (%) | Extreme emotions  n (%) | Emotions interfering in daily life  n (%) | Emotions interfering with treatment  n (%) | Unexplained somatic symptoms  n (%) | At least one symptom  n (%) |
| --- | --- | --- | --- | --- | --- | --- | --- | --- |
| Reference standard: psychiatric diagnostic assessment | | | | | | | |  |
| Oncologist |  |  |  |  |  |  |  |  |
| Adaptive emotions | 97 | 7 (7.2) | 7 (7.2) | 2 (2.1) | 9 (9.3) | 5 (5.2) | 2 (2.1) | 19 (19.6) |
| Maladaptive  emotions | 32 | 10 (31.3) | 7 (21.9) | 3 (9.4) | 10 (31.3) | 4 (12.5) | 3 (9.4) | 18 (56.3) |
| Total | 129 | 17 (13.2) | 14 (10.9) | 5 (3.9) | 19 (14.7) | 9 (7.0) | 5 (3.9) | 37 (28.7) |
| Nurse |  |  |  |  |  |  |  |  |
| Adaptive emotions | 68 | 2 (2.9) | 7 (10.3) | 1 (1.5) | 3 (4.4) | 0 (0.0) | 1 (1.5) | 11 (16.2) |
| Maladaptive emotions | 22 | 7 (31.8) | 8 (36.4) | 1 (4.6) | 6 (27.3) | 1 (4.6) | 1 (4.6) | 12 (54.6) |
| Total | 90 | 9 (10.0) | 15 (16.7) | 2 (2.2) | 9 (10.0) | 1 (1.1) | 2 (2.2) | 23 (25.6) |
|  |  |  |  |  |  |  |  |  |
| Reference standard: patient’s subjective need for professional mental health care | | | | | | | |  |
| Oncologist |  |  |  |  |  |  |  |  |
| Adaptive emotions | 94 | 9 (9.6) | 6 (6.4) | 1 (1.1) | 12 (12.8) | 5 (5.3) | 4 (4.3) | 23 (24.5) |
|  |  |  |  |  |  |  |  |  |
| Maladaptive emotions | 35 | 8 (22.9) | 8 (22.9) | 4 (11.4) | 7 (20.0) | 4 (22.9) | 1 (2.9) | 14 (40.0) |
| Total | 129 | 17 (13.2) | 14 (10.9) | 5 (3.9) | 19 (14.7) | 9 (7.0) | 5(3.9) | 37 (28.7) |
| Nurse |  |  |  |  |  |  |  |  |
| Adaptive emotions | 62 | 3 (4.8) | 6 (9.7) | 1 (1.6) | 3 (4.8) | 0 (0.0) | 2 (3.2) | 12 (19.4) |
| Maladaptive emotions | 28 | 6 (21.4) | 9 (32.1) | 1 (3.6) | 6 (21.4) | 1 (3.6) | 0 (0.0) | 11 (39.3) |
| Total | 90 | 9 (10.0) | 15 (16.7) | 2 (2.2) | 9 (10.0) | 1 (1.1) | 2 (2.2) | 23 (25.6) |

Notes. (1) Patients were categorized as experiencing either adaptive or maladaptive emotions, using two reference standards: (a) psychiatric diagnostic assessment , or (b) patient’s subjective need for professional mental health care. n = number of patients with adaptive or maladaptive emotions, according to the respective reference standard. (2) The percentages are relative to the subgroup (adaptive or maladaptive emotions).
